# The Japanese PCI Registry (J-PCI): 17 Years of Insights Into Quality, Outcomes, and Practice Evolution

**DOI:** 10.1016/j.jacasi.2026.02.005

**Published:** 2026-04-07

**Authors:** Saeko Takahashi, Shun Kohsaka, Kyohei Yamaji, Tetsuya Amano, Ken Kozuma

**Affiliations:** a Department of Cardiology, Shonan Oiso Hospital, Oiso, Japan; b Department of Cardiology, Shonan Kamakura General Hospital, Kamakura, Japan; c Department of Cardiology, Keio University School of Medicine, Tokyo, Japan; d Department of Cardiology, Kyoto University, Kyoto, Japan; e Department of Cardiology, Aichi Medical University, Nagakute, Japan; f Department of Cardiology, Teikyo University Hospital, Tokyo, Japan

**Keywords:** Asia, J-PCI registry, national registry, percutaneous coronary intervention, quality of care

**Figure:**
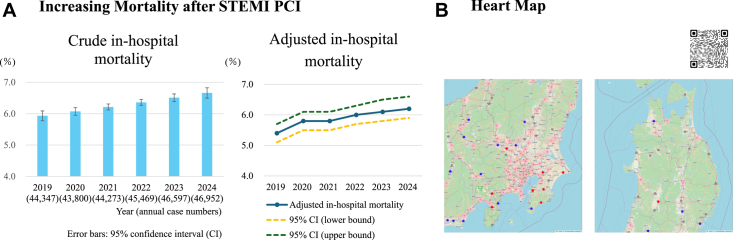

Clinical trials that inform practice guidelines have mainly been conducted in Western populations, whose applicability to Asian patients is limited due to differences in ethnicity, body size, healthcare systems, and bleeding risk. These factors have contributed to distinct percutaneous coronary intervention (PCI) practices in Japan. For example, the Japanese PRASugrel Compared to Clopidogrel For Japanese PatIenTs with ACS Undergoing PCI trial (PRASFIT-ACS trial) used reduced-dose prasugrel (20 mg loading; 3.75 mg maintenance)^1^ compared with the standard dosing in TRITON-TIMI 38 (60 mg loading; 10 mg maintenance).^2^ J-PCI registry data also show that even reduced-dose prasugrel carries higher bleeding risk than clopidogrel, underscoring the need for population-specific evidence.^3^ In the registry, bleeding is classified as major (Academic Research Consortium-2 Types 3-5, Thrombolysis In Myocardial Infarction major) or minor (Academic Research Consortium-2 Types 1-2, Thrombolysis In Myocardial Infarction minimal/minor).^4^^,^^5^

Established in 2007 under the Japanese Association of Cardiovascular Intervention and Therapeutics (CVIT), the J-PCI registry is Japan’s representative national PCI registry.^4^^,^^5^ Its study protocol is approved by an independent ethics committee at Osaka University and adheres to the Declaration of Helsinki. As of 2024, it encompasses more than 247,000 annual PCI cases across 1,189 facilities, covering more than 90% of PCI-performing hospitals.^6^ Data submitted via the National Clinical Database platform include baseline characteristics, procedural details, and in-hospital outcomes. Cases with missing essential variables are excluded according to standard J-PCI analytic practice. At the variable level, the maximum proportion of missing values across all variables was 6.3%.^6^ Each hospital designates a data manager, and annual training is provided to standardize submissions. Participation is required for CVIT board certification and renewal, promoting data completeness and quality. Random audits (∼20 institutions per year) are also conducted. Since 2016, CVIT has solicited national research proposals, resulting in more than 70 published studies. This review summarizes major insights generated by the J-PCI registry and its impact on clinical practice and health policy.

## Major Findings From the Registry

### In-hospital mortality after PCI

In-hospital mortality is the most frequently analyzed outcome among J-PCI studies (Table 1). Overall, in-hospital mortality after PCI in Japan ranges from 0.7%-0.9%.^7^, ^8^, ^9^ Older patients have higher rates of mortality and bleeding, and patients with acute coronary syndrome (ACS) show markedly worse outcomes than those with chronic coronary syndrome (CCS).^10^

**Table 1:** In-Hospital Mortality After PCI

Category	Subgroup	In-Hospital Mortality (%)
Overall PCI	All PCI cases^7^, ^8^, ^9^	0.7-0.9
	Rotational atherectomy^18^	0.6
CCS	PCI for CCS^10^^,^^14^	<1.0
	Age 60-69/70-79/80-89/≥90^10^	0.07/0.09/0.21/0.62
	CCS treated by TRI^14^	0.1
	CCS treated by TFI^14^	0.3
	Dialysis patients^17^	0.2
	UPLMT lesions^16^	0.3
	Without PAD^19^	0.7
	With PAD^19^	1.0
ACS	Primary PCI^10^, ^11^, ^12^, ^13^	1.6-6.0
	Age 60-69/70-79/80-89/≥90^10^	1.22/1.56/2.64/5.18
	ACS treated by TRI^12^^,^^14^	0.6-1.0
	ACS treated by TFI^12^^,^^14^	2.1-2.9
	ACS without CS^13^^,^^14^	0.7-1.3
	STEMI without CS^15^	2.3
	Dialysis patients^17^	3.3
	STEMI with UPLMT lesions^16^	15.0
	ACS with CS^13^^,^^15^	13.2-17.0
	ACS with CS and CA^15^	36.7
ACS with CS required MCS^21^	Impella alone	24.6
	IABP alone	26.1
	VA-ECMO and Impella	46.9
	VA-ECMO and IABP	55.7
	VA-ECMO alone	58.5

ACS = acute coronary syndrome; CA = cardiac arrest; CCS = chronic coronary syndrome; CS = cardiogenic shock; IABP = intra-aortic balloon pump; MCS = mechanical circulatory support; PAD = peripheral artery disease; PCI = percutaneous coronary intervention; TFI = transfemoral intervention; TRI = transradial intervention; UPLMT = unprotected left main trunk; VA-ECMO = venoarterial extracorporeal membrane oxygenation.

Among patients with ACS, in-hospital mortality after primary PCI ranges from approximately 1.6%-6.0%.^10^, ^11^, ^12^, ^13^ Mortality rates have improved over time in the absence of cardiogenic shock (CS), reaching 0.7%-1.3% in ACS,^13^^,^^14^ 2.3% in ST-segment elevation myocardial infarction (STEMI),^15^ and <1.0% in transradial intervention (TRI) cases of ACS.^12^^,^^14^ However, prognosis remains poor in high-risk groups. Mortality reaches 15.0 % in STEMI due to unprotected left main trunk (UPLMT) lesions,^16^ 13.2%-17.0% in ACS with CS,^13^^,^^15^ and >30% with concomitant cardiac arrest (CA).^15^

In contrast, in-hospital mortality among patients with CCS undergoing PCI is generally <1.0%.^10^^,^^14^ However, outcomes vary by presentation and comorbidity. In UPLMT disease, in-hospital mortality rate after PCI for CCS is 0.3%.^16^ Calcified lesions have become an increasing focus with the expanding use of debulking devices, and Japan’s higher prevalence of dialysis further adds procedural complexity, as dialysis patients have higher in-hospital mortality.^17^ In cases requiring rotational atherectomy (RA), mortality is around 0.6% across various indications, including ACS.^18^ Peripheral artery disease (PAD) also becomes a subject of increasing attention. CCS patients with PAD show nearly 50% higher mortality than those without PAD.^19^

### ACS with CS

CS remains the strongest determinant of mortality in ACS, driving increasing reliance on mechanical circulatory support (MCS). Routine use of the intra-aortic balloon pump (IABP) has been downgraded in clinical practice guideline recommendations,^20^ albeit in Japan, its use has continued. Impella became available in 2017, and its use is rapidly increasing.^21^

Among ACS patients with CS, 49.6%-52.9% required MCS.^15^^,^^21^ Between 2019 and 2021, 60.0% of ACS patients who required MCS were treated with IABP alone, 34.9% with venoarterial extracorporeal membrane oxygenation (VA-ECMO), and 5.1% with Impella (Abiomed).^21^ In-hospital mortality was highest with those treated with VA-ECMO alone (58.5%), followed by VA-ECMO in addition to IABP (55.7%), and VA-ECMO in addition to Impella (ECPella; 46.9%). Mortality rate among patients treated with IABP alone and Impella alone was 26.1% and 24.6%, respectively.^21^ Importantly, beyond device selection, timely coronary reperfusion remains the critical determinant of survival in STEMI with CS. Shorter door-to-balloon times are consistently associated with lower mortality.^15^ The overall prognosis of ACS complicated by CS remains poor. However, the use of Impella may offer opportunities to improve outcomes in the future.^22^

### Institutional volume and outcomes

A strong relationship between institutional PCI volume and clinical outcomes has been demonstrated in prior studies.^23^ The J-PCI registry similarly showed that both in-hospital mortality and composite adverse events were significantly higher in hospitals performing fewer than 200 PCIs per year.^8^^,^^9^^,^^13^^,^^16^^,^^18^^,^^24^ Particularly in ACS complicated by CS, in-hospital mortality was inversely related to institutional PCI volume.^13^ For UPLMT PCI, high-volume centers had substantially better outcomes than centers with fewer than 216 PCIs annually.^16^ The lowest rates of in-hospital mortality were observed in high-volume institutions performing more than 488 PCIs per year, compared with lower-volume centers (OR: 0.51; CI: 0.3-0.86; *P* = 0.01).^16^ Institutional experience with RA was also associated with improved results.^18^ Interestingly, coronary artery bypass grafting volume was associated with PCI outcomes in low-volume centers.^9^ This suggests a greater role for surgical backup in these institutions.

In contrast, studies from the J-PCI registry found no clear association between individual operator volume and outcomes.^8^^,^^16^^,^^18^^,^^24^ Recently, we demonstrated that female interventionalists (n = 447; 7.3%) had similar mortality and complication rates, despite performing fewer PCI procedures and a higher proportion of STEMI cases than their male counterparts.^25^

### Urban-rural disparities

In Japan, national insurance coverage helps minimize socioeconomic disparities. However, geographic variation remains a concern, as it continues to affect outcomes. PCI volumes are generally lower in rural areas compared with urban centers. Among patients with ACS, population density was not associated with door-to-balloon time, yet in-hospital mortality was significantly lower in high-density areas (low-density: 2.9%; medium-density: 2.6%; high-density: 2.4%; *P* < 0.001).^26^ The higher mortality observed in rural regions is largely attributable to longer symptom onset-to-balloon times compared with urban settings.

**Figure 1:**
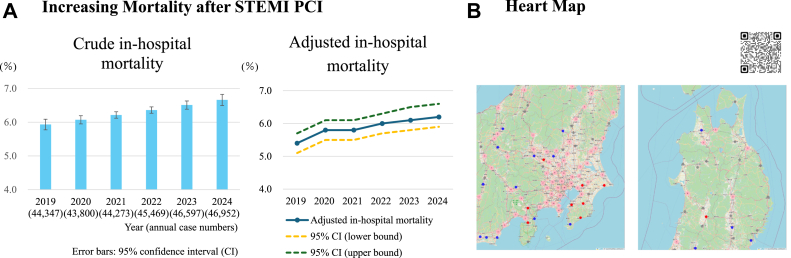
Increasing Mortality After STEMI PCI and Heart Map (A) From 2019-2024, in-hospital mortality among patients with STEMI has increased. This trend likely reflects population aging and a rising proportion of severe presentations such as shock or cardiac arrest. Adjusted in-hospital mortality among STEMI patients was estimated using a multivariable logistic regression model including age, sex, smoking status, hypertension, diabetes mellitus, dyslipidemia, chronic kidney disease, maintenance dialysis, peripheral artery disease, chronic lung disease, prior heart failure, and the presence of cardiac arrest, cardiogenic shock (CS), or acute heart failure within 24 hours before PCI. Corresponding 95% CIs were calculated as Wald-type intervals on the logit scale. (B) The Heart Map, launched on the CVIT website in May 2025, enables users to identify PCI-capable hospitals for ACS. Circles represent the number of hospitals within each region and zooming reveals individual facilities. Blue dots indicate training institutions and red dots indicate affiliated training institutions, categorized by annual PCI volume (<200 vs ≥200 procedures). Clicking on a facility provides additional details, including the number of interventional cardiologists and their certifications. ACS = acute coronary syndrome; CS = cardiogenic shock; CVIT = Japanese Association of Cardiovascular Intervention and Therapeutics; PCI = percutaneous coronary intervention; STEMI = ST-segment elevation myocardial infarction.

Japan has maintained relatively low ACS mortality rates, because PCI is widely accessible across the country. However, mortality among STEMI patients has increased in recent years due to population aging and an increasing proportion of ACS complicated by CS (Figure 1A).^6^ To address these concerns, CVIT introduced the “Heart Map” to support regional care delivery (Figure 1B).^27^ This map allows users to identify hospital locations, CVIT membership, and distribution of certified interventionalists. By selecting an appropriate hospital in advance for symptoms such as chest pain, patients can reduce delays from the onset of ACS to treatment. The system also promotes patient awareness by encouraging proactive decisions to protect one’s own life. Furthermore, visualization of the regional healthcare structure highlights disparities and capacity limitations, with the aim of fostering collaboration among residents and local governments to address healthcare challenges.

### Sex differences in outcomes after PCI

Women undergoing PCI are generally older and have more comorbidities than men.^10^^,^^28^ In our analysis of 562,640 PCI patients, female sex independently predicted in-hospital mortality in both ACS and CCS (ACS: OR: 1.13; 95% CI: 1.04-1.22; *P* = 0.002; CCS: OR: 1.27; 95% CI: 1.02-1.57; *P* = 0.029) and was also associated with higher bleeding risk (ACS: OR: 2.00; 95% CI: 1.76-2.26; *P* < 0.001; CCS: OR: 2.52; 95% CI: 2.16-2.93; *P* < 0.001).^10^ In another analysis of 43,239 patients with non-STEMI undergoing PCI, female sex independently predicted bleeding complications (OR: 1.94; 95% CI: 1.35-2.79; *P* < 0.001),^28^ but not in-hospital mortality (OR: 1.05; 95% CI: 0.79-1.40; *P* = 0.747).^28^ Overall, associations between sex and mortality were modest, whereas the excess risk of bleeding among women was consistent and robust, informing revisions to recent Japanese clinical practice guidelines.^29^^,^^30^

### Unique strategy

Clinical practice in Japan emphasizes procedural precision and bleeding avoidance, shaping the unique PCI strategies described below.

#### Application of “leaving nothing behind” strategy

##### Drug-coated balloon strategy

Drug-coated balloons (DCBs) have expanded the scope of revascularization without stenting and have attracted attention as the “leaving nothing behind” strategy. In ACS patients with a single de novo lesion, outcomes with DCB-only treatment were comparable with those with drug-eluting stents, with similar rates of all-cause mortality (4.1% vs 4.3%), cardiovascular death (2.3% vs 2.3%), and nonfatal ACS (4.1% vs 4.3%) at 1 year.^31^ The high use of intravascular imaging in Japan (>90%) may have contributed to these favorable results.^31^

##### Atherectomy revival

Directional coronary atherectomy (DCA), once widely used internationally, decreased after the introduction of drug-eluting stents but was reintroduced in Japan in 2014, often used in combination with DCB as part of the “leave nothing behind” strategy for ostial, bifurcation, and large-vessel lesions. In recent analysis from J-PCI, DCA accounted for 0.6%-0.9% of PCI procedures. Patients undergoing DCA tended to be younger with fewer comorbidities.^32^^,^^33^ Stentless cases after DCB had comparable in-hospital mortality and complication rates, when referenced to non-DCA cases.^32^ Moreover, 1-year outcomes were similar, including all-cause mortality (1.2% vs 2.5%) and major adverse cardiovascular events (1.9% vs 1.8%).^33^

#### Transradial approach

Two unique studies from the J-PCI registry highlight the benefits of TRI.^12^^,^^14^ In Western countries, where glycoprotein IIb/IIIa inhibitors are widely available, evidence has reinforced the preference for radial access. Although these agents have never been approved in Japan, earlier analysis have demonstrated that TRI was associated with a significantly lower risk for in-hospital mortality (Table 1) and access site bleeding (0.04% vs 0.5%; *P* < 0.001) when compared with transfemoral cases among ACS patients.^12^ Beyond clinical outcomes, TRI has also shown substantial economic advantages. Used in 73.8% of patients, TRI resulted in cost reductions for both ACS and CCS patients,^14^ owing to lower rate of bleeding complications. These findings underscore that TRI not only improves safety but also contributes to sustainable healthcare delivery through significant cost savings.

#### Thrombus aspiration practice

The 2015 American College of Cardiology/American Heart Association/Society for Cardiovascular Angiography and Interventions guidelines downgraded routine thrombus aspiration (TA) in STEMI from a class IIa to class III recommendation for primary PCI.^34^ In Japan, TA remains a class IIb indication, partly because potent antiplatelet or antithrombotic therapies (eg, glycoprotein IIb/IIIa inhibitors) are not available. In a recent analysis from J-PCI, among 282,606 ACS patients undergoing PCI, 83,422 (29.5%) received TA.^35^ Compared with cases without TA, its use was associated with higher procedural success rates. TA was used in over half of STEMI cases (52.9%) without increasing in-hospital mortality.^35^

## Comparison of International PCI Registries and the J-PCI Registry

Although PCI registries from various countries differ in structure and follow-up, all aim to improve PCI quality and safety.^36^ The US CathPCI Registry provides detailed clinical data with 30-day to 1-year outcomes for national benchmarking,^37^ Sweden’s SWEDEHEART study offers nationwide long-term follow-up via linkage with personal identification numbers,^38^ and the United Kingdom’s BCIS-NICOR functions as a mandatory audit with publicly reported, risk-adjusted outcomes.^39^ Korea’s K-PCI supports research and practice monitoring.^40^ In contrast, J-PCI emphasizes detailed procedural data and nationwide in-hospital outcomes. Although Japan’s distinct background of an aging population and a high prevalence of dialysis increase patient complexity, core features of J-PCI such as standardized data collection, policy integration, and nationwide quality oversight may help guide registry development in other Asian countries.

## Future Directions

Although J-PCI is observational and operator-dependent, it provides valuable insights into real-world PCI practice. As PCI techniques evolve and patient complexity increases, the registry must capture more granular data, including long-term and postdischarge outcomes. Integrating J-PCI with other national health databases could enable comprehensive follow-up, while advanced analytics, including artificial intelligence, may help identify high-risk patients, personalize treatment, and predict outcomes. Statistical frameworks that account for between-center heterogeneity may further improve the reliability of long-term estimates. The registry may also serve as a model for developing PCI databases and quality-improvement frameworks across Asia.

## Conclusions

The J-PCI registry demonstrates that rigorous quality oversight can coexist with innovation and personalized care. Its design provides a practical model for countries aiming to build reliable cardiovascular registries. Continued commitment to data transparency and sustainability will ensure that J-PCI remains a foundation for improving PCI outcomes across Asia and beyond.

## Funding Support and Author Disclosures

The authors have reported that they have no relationships relevant to the contents of this paper to disclose.
